# Testing the performance of risk prediction models to determine progression to referable diabetic retinopathy in an Irish type 2 diabetes cohort

**DOI:** 10.1136/bjophthalmol-2020-318570

**Published:** 2021-04-26

**Authors:** John J Smith, David M Wright, Irene M Stratton, Peter Henry Scanlon, Noemi Lois

**Affiliations:** 1 Ophthalmology, Wellcome-Wolfson Institute for Experimental Medicine, Queen's University Belfast, Belfast, Northern Ireland, UK; 2 Centre for Public Health, Queen's University Belfast, Belfast, Northern Ireland, UK; 3 Gloucestershire Retinal Research Group, Cheltenham, UK; 4 Ophthalmology, Gloucestershire Hospitals NHS Foundation Trust, Cheltenham, UK

**Keywords:** eye (Globe), imaging, macula, retina, vision

## Abstract

**Background /Aims:**

To evaluate the performance of existing prediction models to determine risk of progression to referable diabetic retinopathy (RDR) using data from a prospective Irish cohort of people with type 2 diabetes (T2D).

**Methods:**

A cohort of 939 people with T2D followed prospectively was used to test the performance of risk prediction models developed in Gloucester, UK, and Iceland. Observed risk of progression to RDR in the Irish cohort was compared with that derived from each of the prediction models evaluated. Receiver operating characteristic curves assessed models’ performance.

**Results:**

The cohort was followed for a total of 2929 person years during which 2906 screening episodes occurred. Among 939 individuals followed, there were 40 referrals (4%) for diabetic maculopathy, pre-proliferative DR and proliferative DR. The original Gloucester model, which includes results of two consecutive retinal screenings; a model incorporating, in addition, systemic biomarkers (HbA1c and serum cholesterol); and a model including results of one retinopathy screening, HbA1c, total cholesterol and duration of diabetes, had acceptable discriminatory power (area under the curve (AUC) of 0.69, 0.76 and 0.77, respectively). The Icelandic model, which combined retinopathy grading, duration and type of diabetes, HbA1c and systolic blood pressure, performed very similarly (AUC of 0.74).

**Conclusion:**

In an Irish cohort of people with T2D, the prediction models tested had an acceptable performance identifying those at risk of progression to RDR. These risk models would be useful in establishing more personalised screening intervals for people with T2D.

## Introduction

Type 2 diabetes mellitus (T2D) is a global epidemic. In 2019, there were approximately 463 million adults living with diabetes worldwide up from 150 million in 2000; this number is estimated to rise to 700 million^1^ (with an estimated global population of 9.4 billion people) by 2045.^2^ The prevalence of diagnosed diabetes in Ireland increased from 2.2% of the adult population in 1998 to 5.2% in 2015.^3^ The prevalence is higher (8.4%) in adults with type 2 diabetes older than 50 years and higher in men than women.^4^ Diabetic retinopathy and its sight-threatening complications, namely diabetic macular oedema (DMO) and proliferative diabetic retinopathy (PDR), which occur in ~7% of people with T2D,^5^ greatly increase the individual’s subsequent risk of blindness and visual impairment.^6^

Screening of the diabetic population by digital fundus photography has the aim of early detection of complications of DR with the intention of providing timely treatment and, thereby, reducing visual loss and blindness in society. The Wisconsin Epidemiologic Study of Diabetic Retinopathy group found that up to 3% of people diagnosed with diabetes after age 30 years will have high risk DR features (such as clinically significant macular oedema) by the time they are first diagnosed with diabetes.^7^ Most international guidelines recommend annual screening for people with diabetes and no or mild DR; repeated examinations at 6-month intervals for those with moderate DR; and referral to an ophthalmologist for people with referable DR (RDR), including those with higher stages of DR (pre-proliferative DR) or its complications (ie, maculopathy and/or PDR).^8–11^

Screening procedures in Europe range widely from annual or biennial systematic photographic screening to non-systematic (opportunistic and ad hoc) screening,^12^ as it was clear that annual appointments for examination with an ophthalmologist for everyone with diabetes was unachievable.^13^ In recent years, with the rapidly increasing prevalence of T2D, an enormous healthcare capacity problem has arisen demanding re-evaluation of the recommended screening strategy.^14^ In the early years of this century, the possibility of extending screening intervals for those individuals determined to be at low risk of progression was entertained.^15^ Factors determining risk of progression from no/minimal DR to RDR are not firmly established and vary among populations.^16–22^ In an Irish population, elevated HbA1c, systolic blood pressure and triglyceride levels as well as prior retinopathy grading were important risk factors determining progression to RDR.^19^ By incorporating data from retinopathy status and also from a systemic assessment, it may be possible to better predict risk and, thereby, personalise screening.^16^ By this means, people at low risk could be seen less frequently, freeing capacity to increase the frequency of screening of those at higher risk and ensuring the sustainability of existent screening programmes.

A variety of risk prediction models for progression to various degrees of vision-threatening DR have been developed and validated in some populations.^23^ Herein, we used data from a prospective cohort study of people with T2D undertaken in Ireland to evaluate the performance of two of these models in this Irish population.

## Participants and methods

The Diabetes Watch Programme, developed by the public health service in Ireland, offered systematic care to people with T2D ≥18 years of age registered in 20 general practitioners’ practices in four counties in the North East region of Ireland. Diagnosis of diabetes and routine systemic assessments adhered to established methods. Details of this screening programme have been provided previously.^19^ In brief, from February 2005 until December 2007, 1265 individuals with pre-existent T2D were recruited to the Diabetes Watch Programme. Subsequently, between February 2008 and July 2013, targeted screening using the FINDRSIC questionnaire^24^ identified a further 1505 newly diagnosed T2D individuals for a total cohort of 2770 people. The cohort, thus, was composed of a combination of prevalent and incident cases. Systemic evaluations including systolic and diastolic blood pressure (BP), HbA1c, serum lipids levels and body mass index (BMI) were undertaken at 4-month intervals. Screening for any DR and RDR using digital retinal imaging was done annually.

Data from the Diabetes Watch Programme was used to test the performance of two prediction models for identification of sight-threatening DR and RDR developed in Iceland^16^ and Gloucester,^22^ respectively. The Icelandic model determines risk and provides a recommended interval for next screening based on retinopathy grading, type and duration of diabetes, HbA1c (or mean blood glucose) and BP. The Gloucester model has three variants, one using retinopathy grades at two screening episodes only, another using, in addition, HbA1c and total serum cholesterol levels, and a third using results of one screening episode, duration of diabetes, and HbA1c and total serum cholesterol levels. To facilitate comparison of accuracy of prediction of progression to RDR between the two models, the final stage of the Icelandic algorithm, where a safe time interval before next screening episode is determined, was omitted. Risk scores were determined and time-dependent receiver operating characteristic (TDROC) curves produced. TDROC curves were used as it is not known at which point between two screening episodes progression to RDR took place.^25^

**Figure 1:**
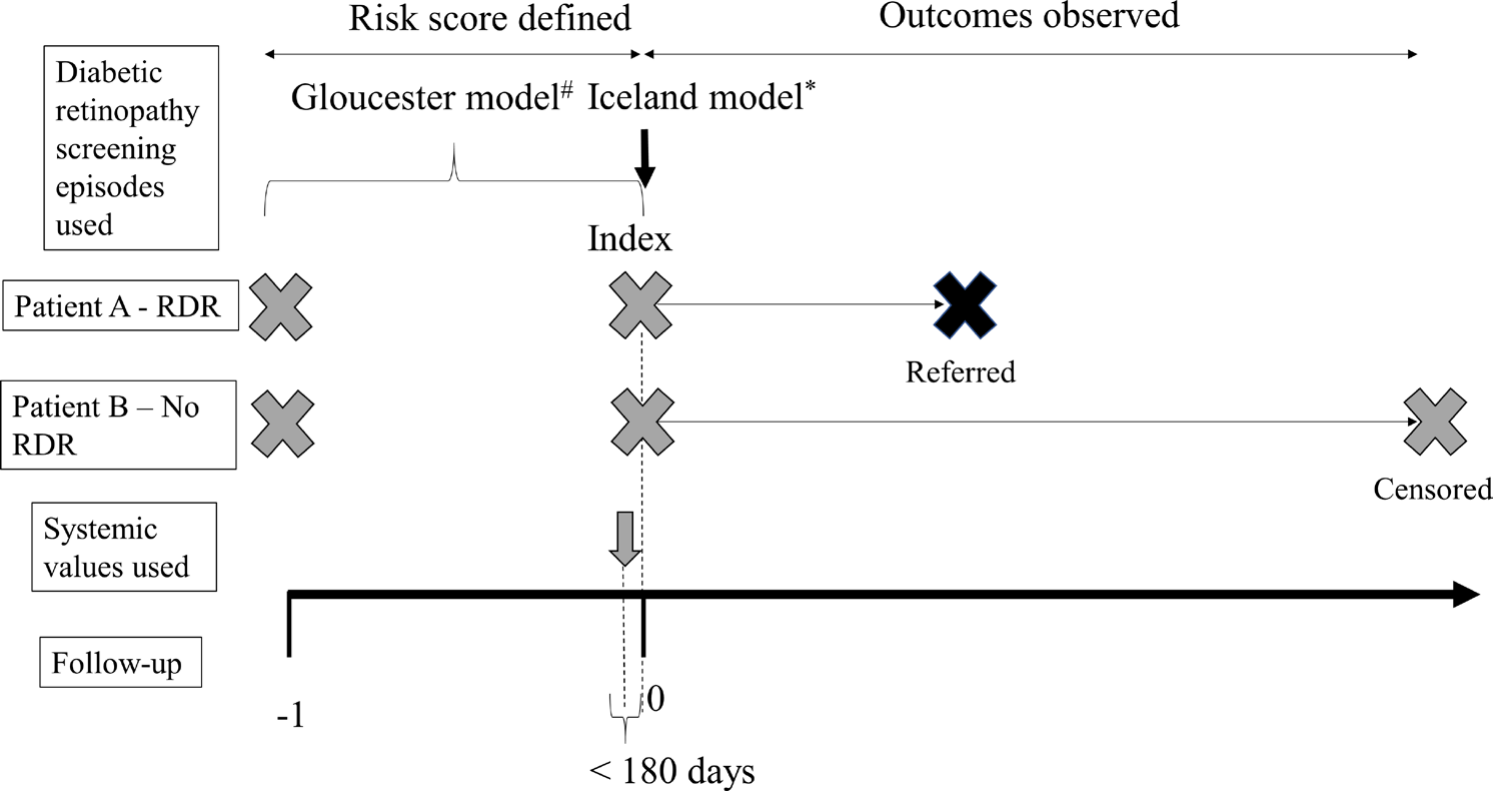
Scheme summarising the methodology followed for data analysis. The Gloucester model required two annual retinal assessments preceding the time to event analysis (index and index−1). The Icelandic model requires a single annual retinal assessment. The models use systemic variables; in this study, values of systemic variables were obtained at the visit immediately before the index screening (within 180 days prior to this visit). Patient A and patient B appear as examples. RDR, referable diabetic retinopathy.

For a valid comparison between prediction models, the cohort and follow-up periods had to be identical for both algorithms. Inclusion criteria and the follow-up periods were determined by the more restrictive of the two models (the Gloucester algorithm), the key criterion being that patients had to have at least three retinal screening episodes, two to determine the risk group and a third to determine the outcome. The first two recorded screening episodes were used to define the risk groups for the Gloucester model; for the Icelandic model, only the second screening was required for the prediction. We refer to the second recorded retinal screening episode as the ‘index’ screening, after which follow-up commenced for both models. Measurements of systemic variables were drawn from the most recent visit prior to the index screening at which a full set of measurements for the variables used by the algorithms was taken (figure 1).

**Figure 2:**
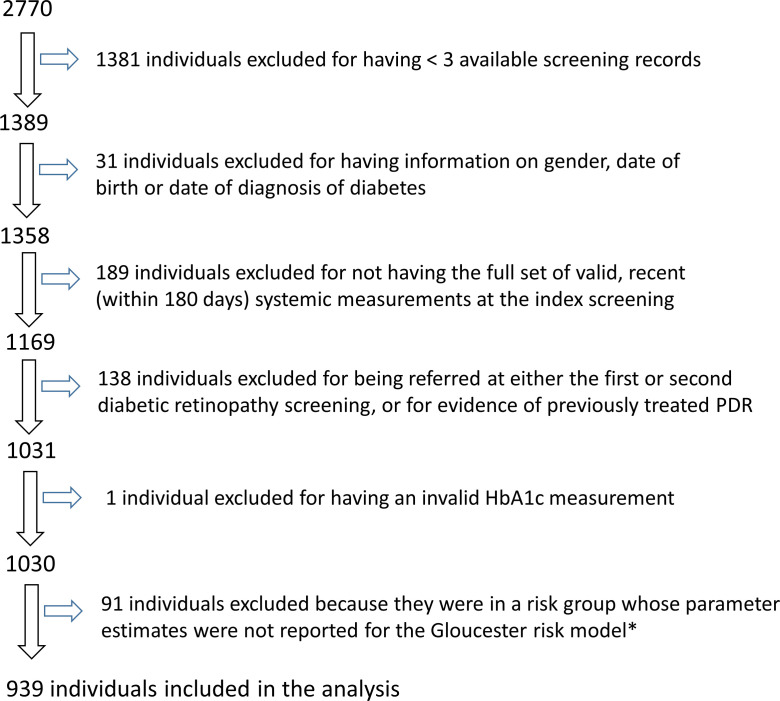
Diagram demonstrating how the cohort of patients (n=939) for the analysis presented in this study was drawn from the whole cohort of the Diabetes Watch population (n=2770). *The 91 individuals were in groups B=R1 in the first screening in one eye and R0 in both eyes in the second screening and C=R1 in the first screening in both eyes and R0 in both eyes in the second screening.

Retinopathy results from the Diabetes Watch Programme were available at an individual eye grading level; grading was a quality-assured consensus of at least two graders, done prospectively. Systemic measurements were masked from those grading the progression outcome (RDR). RDR was defined as R1M1 (ie, presence of maculopathy), R2M0 (ie, presence of pre-proliferative DR), R2M1 (pre-proliferative DR and concomitant maculopathy), R3M0 (PDR) or R3M1 (PDR and concomitant maculopathy) in either eye. Patients were included in the cohort only if they were within one of the risk groups defined by Stratton *et al* 17 having one of three categories of DR at both of the first two eye screenings (index−1, index): (1) no DR in either eye; (2) mild DR in only one eye; (3) mild DR in both eyes. Individuals with other combinations of screening results were excluded, as were those in two of the risk groups (B and C; group B=R1 in the first screening in one eye and R0 in both eyes in the second screening; group C=R1 in the first screening in both eyes and R0 in both eyes in the second screening) where DR was present at the first screen but not at the second. These groups were not included in one of the variants of the Gloucester model reported in Scanlon *et al* 22 and so risk predictions could not be made. Figure 2 summarises the cohort construction.

Follow-up extended from the date of the index screening episode to either the date of referral or, for those not referred (ie, not developing RDR), to the date of the last recorded screening episode, at which remaining patients were censored (figure 1).

### Statistical analysis

ROC analysis of the prognostic capacity of the tested algorithms was performed using the *pROC* and *tdROC* packages in the statistical program *R*. Risk scores were calculated using the three candidate algorithms and TDROC curves fitted predicting outcomes at 2 years post-index screen; 95% CIs for AUC values were obtained using 100 bootstrap samples. Predictions across the entire follow-up period were also assessed using TDROC curves. By this means, performance of the models was evaluated on the Irish cohort data, with the input being risk score and the outcome being whether development of RDR during follow-up was observed in the cohort population.

Risk score calculations were undertaken as described by the authors of the models in their original papers. In the Icelandic model, this merely involved substituting the values observed in the Irish cohort into their model. A survival score was calculated at each index screening (time *t* years since diabetes diagnosis) by first taking a linear combination of the published coefficients from Aspelund *et al* 16 and the corresponding variables in the Diabetes Watch dataset at the last systemic visit before the index screening. The linear combination was then exponentiated and multiplied by the baseline survival probability to give the survival probability *S*(*t*). The individual hazard at time *t* was approximated by *risk*(Δ*t*|disease free at *t*)=1−*S*(*t*+Δ*t*)/*S*(*t*) where Δ*t*=1 month. Thus, relevant values from the Diabetes Watch dataset were substituted into the equations in Aspelund *et al*.^16^ The hazard was multiplied by 100 for use as the individual risk score because AUC calculations were unstable when the probability scale was used.

For the Gloucester models to be applied to the Irish data, individual risk scores were calculated by taking a linear combination of the published coefficients drawn from Table 15A–C (p.24) of Scanlon *et al* 22 and the corresponding variables in the Irish dataset, as recorded at the last systemic visit before the index screening episode. Examples of these calculations are given in Scanlon *et al* (p.16).^22^ Using these risk scores and incorporating the values from the Irish data a direct comparison between the different models was undertaken.

## Results

The analysis cohort consisted of 939 individuals (figure 2) followed for a total of 2929 person years, with a total of 2906 screening episodes (excluding the screening episodes used to define the risk scores). Characteristics of the study cohort included here and those of the entire Diabetes Watch Programme cohort are shown in table 1.

**Table 1:** Patient characteristics of the cohort modelled in the analysis (n=939) and in the entire Diabetes Watch population (n=2770)

Patient characteristics	Modelled cohort n=939	Entire DW cohort n=2770
**Gender**	**Number (%)**	**Number (%)**
Male	534 (56.9)	1588 (57.3)
Female	405 (43.1)	1137 (41.0)
Gender unknown	0 (0)	45 (1.7)
**Baseline retinal assessment (ETDRS equivalent)**		
R0 (10) (both eyes)	817 (87.0)	1750 (63.2)
R1M0 (14 to 35) (either eye)	122 (13.0)	247 (8.9)
Other grade or not available	0 (0)	773 (27.9)
	**Mean (range)**	**Mean (range)**
Age (years)	64 (30–89)	63 (17–108)
Duration of diabetes (years)	3 (0–45)	0 (0–72)
	**Mean (SD) (n missing)**	**Mean (SD) (n missing)**
HbA1c (%)	6.8 (1.2) (0)	7.2 (1.6) (149)
HbA1c (mmol/mol)	50.8 (12.7) (0)	55.6 (17.8) (149)
BMI (kg/m^2^)	31.1 (6.4) (64)	31.0 (6.3) (228)
HDL (mmol/L)	1.2 (0.4) (1)	1.3 (1.1) (142)
LDL (mmol/L)	2.4 (0.8) (2)	2.7 (1.1) (140)
Triglycerides (mmol/L)	3.2 (2.0) (2)	3.6 (2.9) (159)
Diastolic BP (mm Hg)	78 (9.6) (5)	79 (9.6) (90)
Systolic BP (mm Hg)	136 (16.4) (0)	137 (18.0) (98)

Characteristics of the entire Diabetes Watch Cohort were from the first systemic visit (and diabetic retinopathy screening episode, if retinopathy grade was available). The characteristics for the modelled cohort were from the index screening.

BMI, body mass index; BP, blood pressure; DW, Diabetes Watch; HDL, high-density lipoprotein; LDL, low-density lipoprotein; R0, no diabetic retinopathy on fundus images; R1M0, mild non-proliferative diabetic retinopathy and no maculopathy.

The cohort was followed for a median of 3 years (range 0.4–6.4 years; 80 patients were followed up for ≤11 months; 6 for ≤9 months and only 1 for ≤6 months). There were 40 referrals for RDR, encompassing referrals for diabetic maculopathy/pre-proliferative DR (R1M1, R2M0, R2M1) and proliferative DR (R3M0, R3M1).

**Figure 3:**
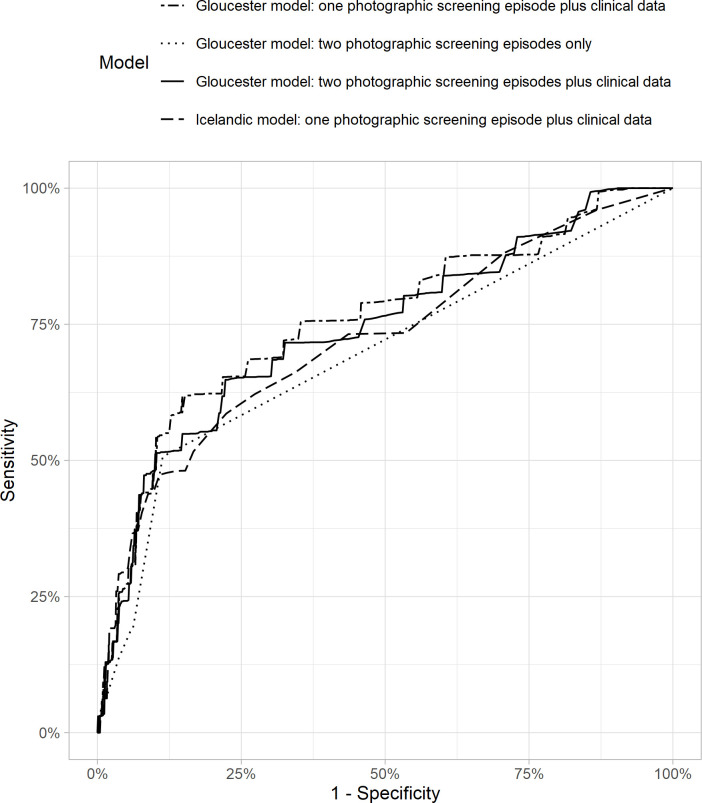
Receiver operating characteristic curves for the Icelandic and Gloucester models.

Icelandic and Gloucester models performed similarly in terms of discrimination at 2 years post-index screening (figure 3), with an AUC of 0.72 (95% CI 0.61 to 0.81) for the Icelandic model; 0.69 (95% CI 0.61 to 0.77) for the Gloucester model that includes only grading of DR at two consecutive screening episodes; 0.74 (95% CI 0.64 to 0.85) for the Gloucester model that includes, in addition, HbA1c and total cholesterol; and 0.76 (95% CI 0.65 to 0.85) for the model that includes retinopathy grading results from one screening episode, HbA1c, total cholesterol and duration of diabetes. Predictions for both models were also similar when estimated across the entire follow-up period, using standard time-independent ROC curves (AUC of 0.74 (95% CI 0.65 to 0.83)) for the Icelandic model; 0.69 (0.61 to 0.77) for the Gloucester model that includes only grading of DR at two consecutive screening episodes; 0.76 (0.67 to 0.84) for the Gloucester model that includes, in addition, HbA1c and total cholesterol; and 0.77 (0.69 to 0.85) for the Gloucester model that includes DR grading from one screening episode, HbA1c, total cholesterol and duration of diabetes.

Considering the entire observation period, for all three models, rates of referral were higher among those with higher risk scores. For example, referral rates among those with Icelandic risk scores in the highest risk quintile were eight times greater than those in the lowest risk quintile (online supplemental figure 1). A similar pattern was seen for the Gloucester models including HbA1c, total cholesterol and duration of diabetes (one screening variant only) (online supplemental figures 23), with a 10-fold difference between lowest and highest risk groups.

10.1136/bjophthalmol-2020-318570.supp1 Supplementary data

10.1136/bjophthalmol-2020-318570.supp2 Supplementary data

10.1136/bjophthalmol-2020-318570.supp3 Supplementary data

The Gloucester model based on screening results alone was dominated by risk group A (R0 in both eyes at the initial and index screenings); there were few individuals in the other risk groups and, thus, there was inadequate power with which to estimate differences among risk groups (table 2).

**Table 2:** Risk predictions for the different risk groups as determined by the Icelandic and Gloucester models and referable diabetic retinopathy (RDR) events observed in the Diabetes Watch programme cohort during the study period

Model	Risk group	Number in risk group	Number of events	Rate of progression to RDR (per 1000 PYs)	Exposure time (PYs)
Icelandic (score quintile)	1	188	3	5.6	537
	2	188	3	5.2	581
	3	188	5	8.3	601
	4	188	6	9.4	636
	5	187	23	40.0	576
Gloucester model using 2 DR gradings only (risk groups)	A	819	20	7.7	2585
	D	58	13	80.0	162
	E	20	2	32.8	61
	F	4	0	0.0	11
	G	20	3	44.4	68
	H	7	0	0.0	21
	I	11	2	88.2	23
Gloucester model using 2 DR gradings and systemic variables and diabetes duration (score quintile)	1	188	2	3.4	582
	2	188	4	7.3	546
	3	188	5	8.8	570
	4	188	10	16.0	626
	5	187	19	31.3	606
Gloucester model using 1 DR grading and systemic variables (score quintile)	1	188	2	3.6	557
	2	188	4	7.1	562
	3	188	4	6.9	577
	4	188	6	9.3	649
	5	187	24	41.1	584

For completeness, group B=R1 in the first screening in one eye and R0 in both eyes in the second screening; group C=R1 in the first screening in both eyes and R0 in both eyes in the second screening.

Risk groups A=no DR in both eyes in first and second screenings; D=no DR in first screening, background DR in second screening in one eye; E=background DR in one eye in first and second screenings; F=background DR in both eyes in first screening, background DR in one eye only in second screening; G=R0 in both eyes in first screening; R1 in both eyes in second screening; H=background DR in one eye in first screening, background DR in both eyes in second screening; I=background DR in both eyes in first and second screenings.

PY, patient-year.

## Discussion

Using data from a longitudinal cohort of people with T2D established in Ireland, we found the risk prediction models tested, developed in Iceland^16^ and Gloucester,^22^ had an acceptable performance^26^ with an AUC of ~0.70 or above, indicating there would be a >70% probability that a randomly selected subject from the screening cohort who developed RDR would have been allocated to the higher risk score category.

We chose to test the Icelandic and Gloucester prediction models given that a recently conducted systematic review^23^ found they performed best overall from the prediction models evaluated. We decided to determine predictions at 2 years as it is unlikely screening intervals will be extended, initially, beyond that period, given the concern that extending intervals further may have an impact on attendance. We, however, present also predictions across the entire follow-up period which, reassuringly, produced similar results. The accuracy of the predictions in the Irish population of the models tested seemed to be somewhat less than that found in other studies.^16 22 23^ This may relate to intrinsic differences in the populations tested or a limitation related to the smaller cohort and small number of people converting to RDR in the current study.

Although initially planned as an annual occurrence for people with diabetes, the Icelandic DR screening programme moved to biennial screening for those with no evidence of DR in 1994.^27^ It is likely that biennial screening will be introduced soon also in UK and Ireland for people at low risk of progression to RDR. How to identify low-risk and high-risk groups for this purpose is debatable and an area of active research currently.

The concept of a prediction model forecasting DR progression was formally proposed in Iceland.^16^ The Icelandic model used as input variables the individual eye retinopathy grading, as defined by the ETDRS classification system, alongside information on type and duration of diabetes, HbA1c values and systolic blood pressure with the output being the development of sight-threatening diabetic retinopathy (STDR) (ie, DMO and/or PDR). The algorithm, which is available as an app,^28^ provides a recommended time for next screening, making it both practical and simple to use, provided that there is access to all required information, including HbA1c and BP values. The validity of this algorithm has been shown in different populations from Denmark,^16^ Spain,^29^ Netherlands,^30^ UK^31^ and now Ireland, with AUC values varying from 0.74^29^ for a mixed population of T1D and T2D (86.6% T2D) to 0.80^31^ and 0.83^30^ for people with T2D exclusively. Accepting a 2-year ceiling on screening interval, a 40% reduction in screening frequency would be achievable using this algorithm.^30 31^ In our Irish population, this algorithm performed adequately, despite the fact that it was originally created to predict risk of STDR and we used it to predict risk of RDR.

Three algorithms developed by the Gloucester group were tested herein: one using only results of DR screening at two consecutive yearly visits; another using, in addition, HbA1c and total cholesterol levels; and a third using results from a single DR screening, HbA1c, total cholesterol and duration of diabetes. From a practical perspective, requiring HbA1c, lipid or BP levels to estimate risk complicates matters by requiring the DR screening programmes to have a link between retinopathy grading and measures of systemic parameters, currently not in place. As suggested previously,^17^ stratifying people based on presence/absence of mild NPDR in one or both eyes at first and/or second DR screening may be all that is required to identify those at very low risk of progression to RDR, in whom the screening interval could be extended, and could be easily implemented by screening programmes. This would reduce the burden of screening for DR and should be cost-saving. The results presented herein, however, suggest that adding systemic parameters to the model may increase the accuracy of the prediction (with improved predictions from an AUC of 0.69 when retinopathy grading alone was used to 0.74 when HbA1c and cholesterol were added as per the Gloucester models). Similarly, using a single retinopathy grade but including HbA1c, cholesterol and duration of diabetes improve the prediction (although not statistically significantly), with an AUC of 0.76.

With the exception of the screening-only Gloucester model, each of the risk models applied here included both retinopathy grade and systemic variables as potential predictors. These variables should not be considered independent risk factors as, for example, HbA1c is likely to influence both the existing retinopathy grade and the progression to RDR. Therefore, it would be inappropriate to interpret the estimated coefficients for these variables within a causal framework. However, the models were formulated primarily to make the best use of available data to generate risk predictions and are applicable for this purpose in this study despite this caveat

Given that annual screening for people with low risk is not cost-effective^22^ and considering pressures related to capacity faced by screening programmes, the move towards extended screening for those at low risk is now almost inevitable. A recently published study^32^ showed that personalised screening for DR using the Icelandic prediction model^16^ would be cost-saving, but it has been estimated that it would increase by ~11% the number of cases with delayed diagnosis of STDR. It should be noted, however, that the repercussion of the delayed diagnosis in these cases is unclear, as this was not investigated (ie, it is uncertain whether the delayed diagnosis in these cases would have led to permanent visual loss). Furthermore, it should be noted that in this study, ‘grade 4’ (‘photocoagulated retinopathy’) was included in the definition of STDR. People with panretinal photocoagulation, if stable, are not at risk of sight loss and, even if active, would be expected to be at a lesser risk of sight loss than patients that are treatment-naïve. On this regard, a recently conducted randomised controlled trial demonstrated that individualised screening based on demographics, DR screening and clinical data was non-inferior to annual screening, and it was safe and cost-effective.^33^

Existing models predict development of STDR or RDR. STDR encompasses DMO and PDR and RDR, in addition, pre-proliferative DR. The impact of missing one or another is expected, however, to be quite different. DMO does not cause rapid visual loss whereas PDR can do. DMO, untreated, would lead to reduced central vision whereas PDR can lead to full blindness, even to the loss of an eye. In contrast to PDR (unless advanced PDR with vitreous haemorrhage and/or tractional retinal detachment ensues), DMO affects central vision and, thus, it is more likely that its symptoms may be recognised by patients, whereas PDR develops asymptomatically. Importantly, systemic risk factors have been shown to contribute differently to the development of DMO and PDR^34^ and, thus, it would seem essential that predictions of risk are estimated separately for each of these DR complications.

An unresolved but important matter is the determination of the level of prediction (AUC) which would be satisfactory and acceptable for an algorithm to be used in clinical practice.

Strengths of this study include the use of observational data from routine clinical practice, relatively long follow-up, standardised criteria for referral and the quality-assured retinopathy grading. When reporting this study, we follow the TRIPOID guidelines for prediction model validation.^35^ The strict criteria we used to construct the cohort of patients in which we tested the different prediction models meant we lost some of the population of the entire Diabetes Watch cohort, but it meant also that all models were tested in the exact same people, making comparisons among models fair. Limitations include the relatively small cohort of patients eligible for the testing of the algorithms and the small number of patients that developed RDR during the follow-up period. It is possible that RDR could have occurred in people lost to follow-up, which was not accounted for in the results presented herein. It should be noted that the great majority of people in the cohort used in this study had no DR in either eye (87%) when entering the programme, and this should be taken into consideration when interpreting the data presented.

## Data Availability

Data are available on reasonable request to NL, The Wellcome-Wolfson Institute for Experimental Medicine, Queen’s University, Belfast (orcid.org/0000-0003-2666-2937).
