# Supplementary figures and images for: Testing the performance of risk prediction models to determine progression to referable diabetic retinopathy in an Irish type 2 diabetes cohort
Source: Br J Ophthalmol. 2021 Apr 26;106(8):1051–6. doi: 10.1136/bjophthalmol-2020-318570 (PMC9340042; doi:10.1136/bjophthalmol-2020-318570)

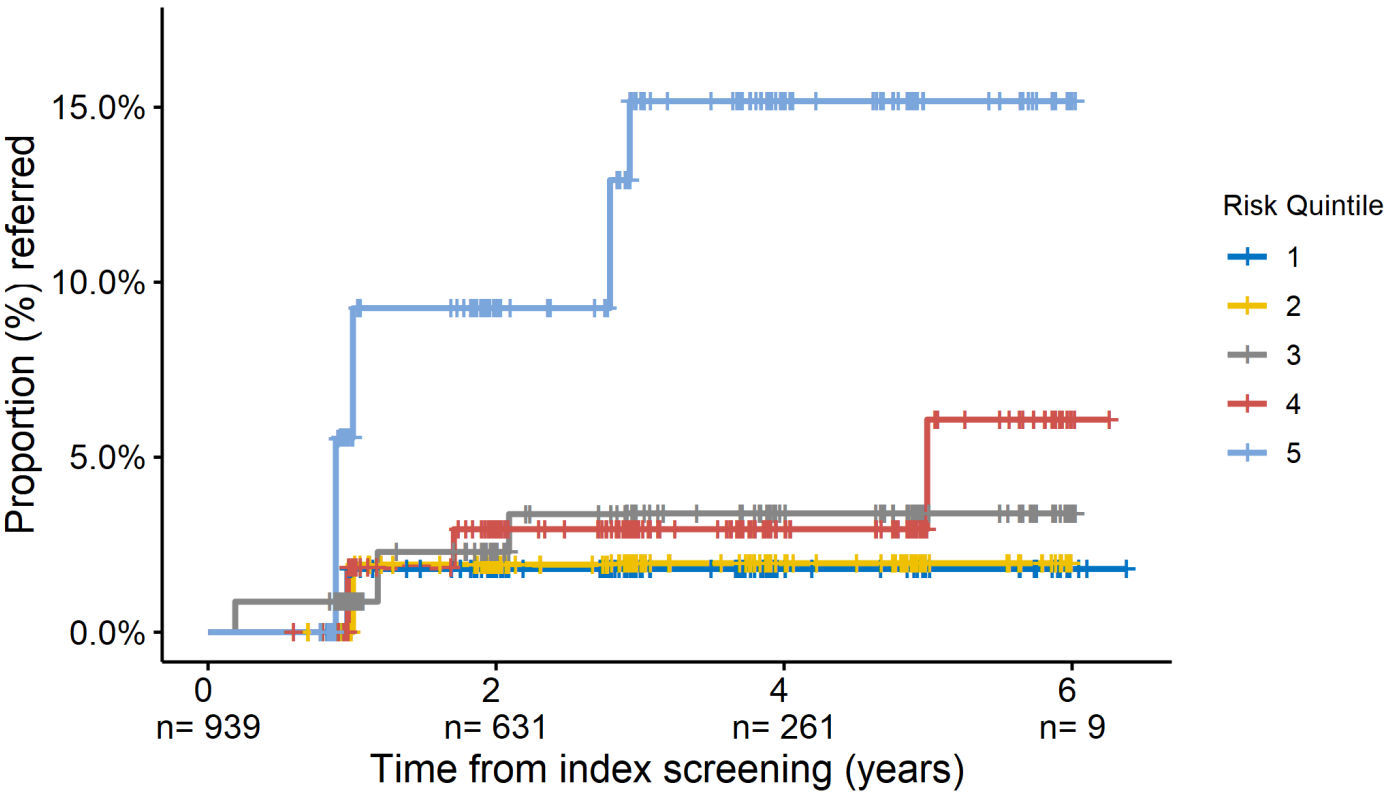

Supplement: Supplementary data [file bjophthalmol-2020-318570supp001.pdf]

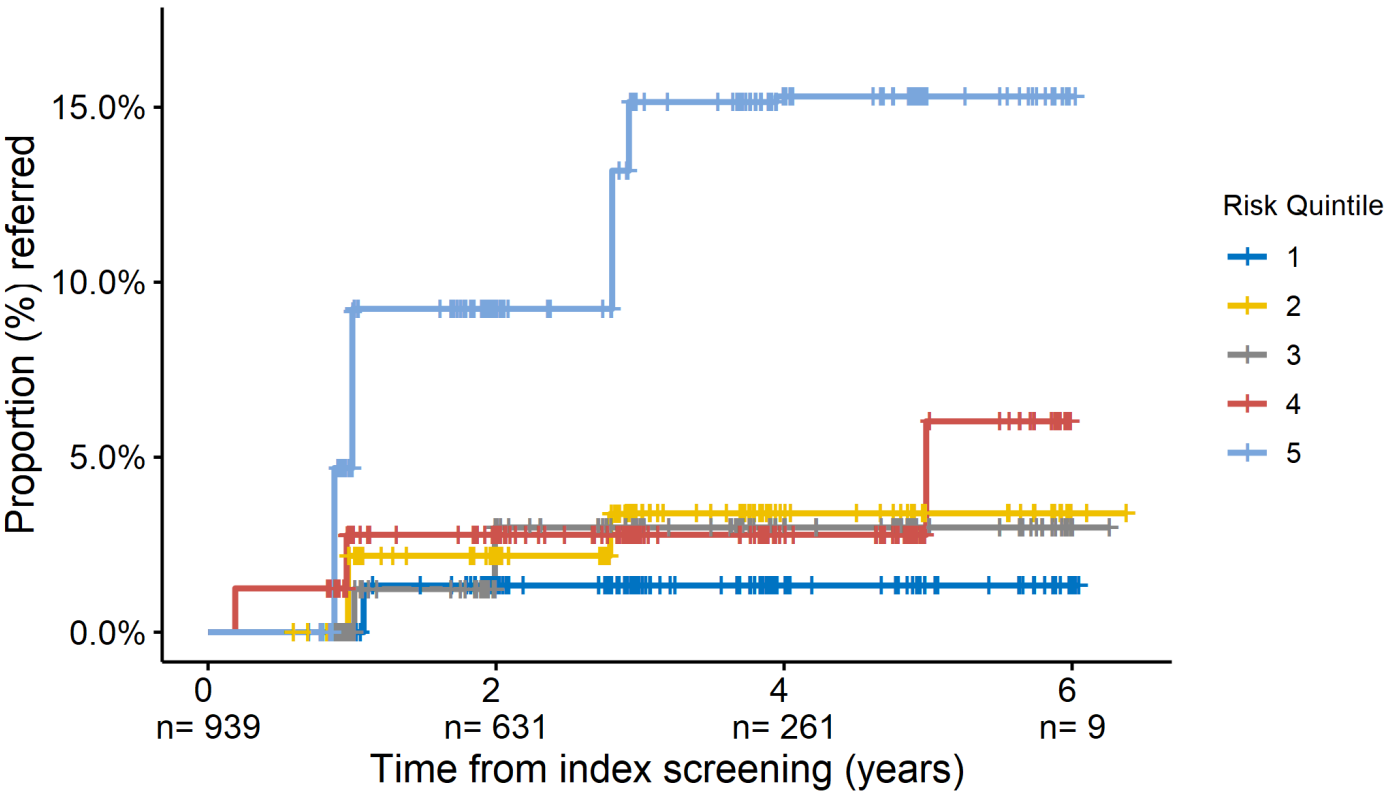

Supplement: Supplementary data [file bjophthalmol-2020-318570supp002.pdf]

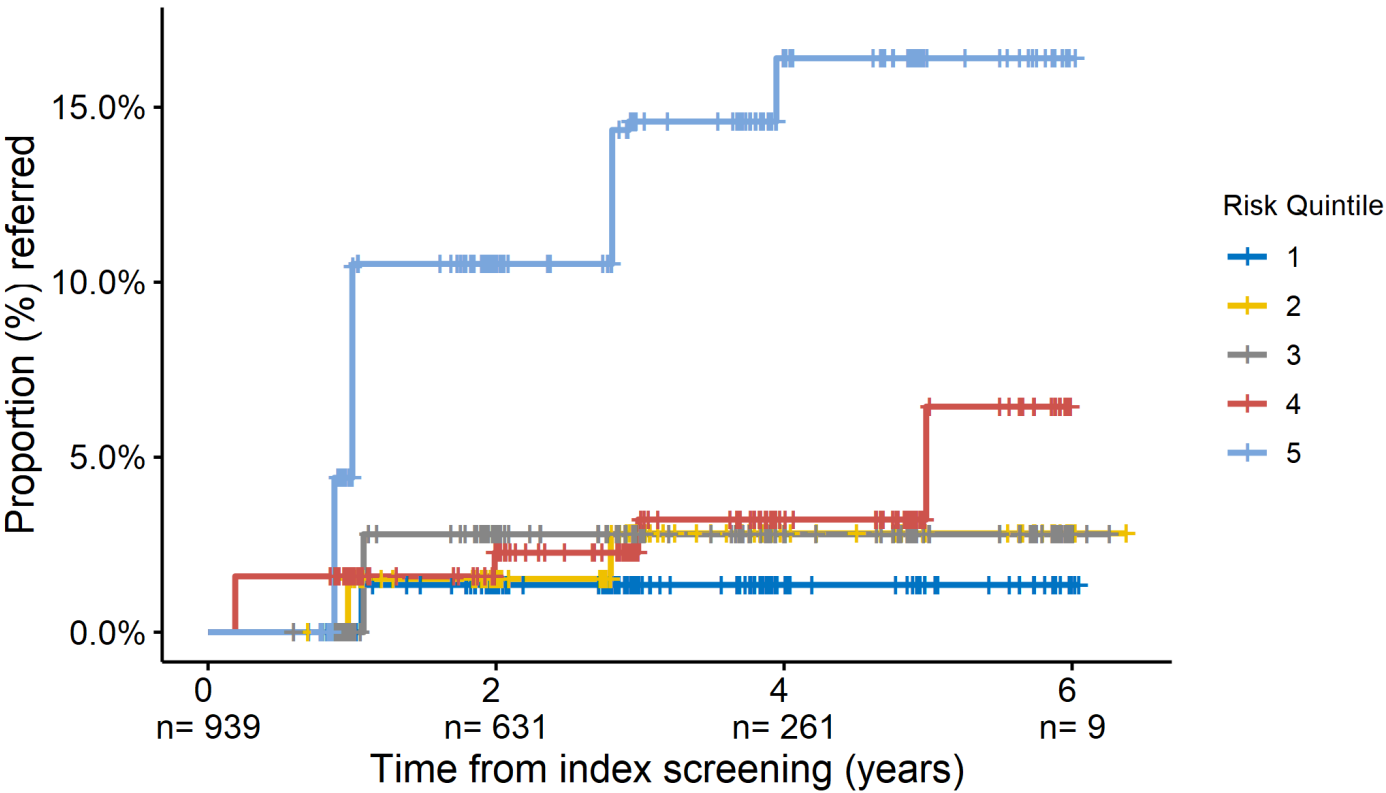

Supplement: Supplementary data [file bjophthalmol-2020-318570supp003.pdf]
